# Topographic analysis of macular choriocapillaris flow deficits in diabetic retinopathy using swept–source optical coherence tomography angiography

**DOI:** 10.1186/s40942-020-00209-0

**Published:** 2020-03-19

**Authors:** Isaac Gendelman, A. Yasin Alibhai, Eric M. Moult, Emily S. Levine, Phillip X. Braun, Nihaal Mehta, Yi Zhao, Akihiro Ishibazawa, Osama A. Sorour, Caroline R. Baumal, Andre J. Witkin, Elias Reichel, James G. Fujimoto, Jay S. Duker, Nadia K. Waheed

**Affiliations:** 1 New England Eye Center, Tufts Medical Center, 260 Tremont St, Boston, MA 02116 USA; 2 Tufts University School of Medicine, Boston, MA USA; 3 Yale University School of Medicine, New Haven, CT USA; 4 Warren Alpert Medical School of Brown University, Providence, RI USA; 5 Department Electrical Engineering and Computer Science, Massachusetts Institute of Technology, Cambridge, MA USA; 6 Friedman School of Nutrition Science and Policy, Tufts University, Boston, MA USA; 7 Department of Ophthalmology, Asahikawa Medical University, Asahikawa, Japan; 8 Department of Ophthalmology, Tanta University, Tanta, Egypt

**Keywords:** Choriocapillaris, Diabetic retinopathy, Imaging, OCT angiography, Ophthalmology, Retina

## Abstract

**Background:**

The purpose of this study was to investigate the association between diabetic retinopathy (DR) severity and macular choriocapillaris (CC) flow deficit percentage (FD %) in different macular regions using swept-source optical coherence tomography angiography (SS-OCTA).

**Methods:**

Diabetic patients with SS-OCTA images were graded by severity and retrospectively assessed. CC FD % was calculated in four different regions of the OCTA image: inner, middle, outer, and full-field region. The generalized estimating equations (GEE) approach for clustered eye data was used to determine effect size and significance of age and disease severity on FD % for each region.

**Results:**

160 eyes from 90 total diabetic patients met inclusion criteria. Out of 90 patients, 33 had no DR, 17 had mild nonproliferative DR (NPDR), 8 had moderate NPDR, 10 had severe NPDR and 22 had proliferative DR. Age and DR severity had a significant positive association with FD % for each region studied with a greater effect in the two centermost regions. The increase in flow deficit percentage per year of age by region was: inner 0.12 (p < 0.001), middle 0.09 (p < 0.001), outer 0.05 (p < 0.001, full-field 0.06 (p < 0.001). The increase in flow deficit percentage per increase in diabetic retinopathy severity stage by region was: inner 0.65 (p < 0.0087), middle 0.56 (p < 0.0012), outer 0.33 (p < 0.045), full-field 0.36 (p < 0.018).

**Conclusions:**

Topographic analysis of the CC FD % in diabetic eyes suggests that CC flow impairment corresponds to DR severity, with all studied regions of the CC significantly affected. There was greater regional impairment due to age and disease severity in the inner and middle regions.

## Background

Diabetic retinopathy (DR) is the most common microvascular complication of diabetes, and its diagnosis and treatment are critical to ophthalmic practice [1, 2]. In addition to retinopathy, diabetic choroidopathy has also been recognized as part of the vascular inflammatory effect of diabetes [3, 4]. Prior studies show that diabetic choroidopathy can be assessed clinically with optical coherence tomography (OCT), demonstrating decreased choroidal thickness in diabetic patients as well as decreased capillary perfusion density in the choriocapillaris (CC) [5–8]. The ability to analyze the choroid, and specifically the choriocapillaris using OCT angiography (OCTA), has led to new descriptions of the pathophysiologic effects of diabetes [9, 10].

The CC is a single layer of capillaries that supplies the retinal pigment epithelium and photoreceptors [11]. It is a dense network that is unique among capillary beds in its increased caliber and permeability [12]. The recent increased interest in the choriocapillaris results directly from advances in OCTA, a technology that uses OCT motion signal to image depth-resolved blood flow. Previous studies of the CC were limited to post-mortem histology as other methods of imaging the vasculature, such as fluorescein or ICG angiography, cannot easily visualize the small, fenestrated vessels of the CC [13]. In addition, OCT imaging of the CC has historically been challenging because of the signal scattering effect of the RPE [11]. New technologies in OCT such as swept-source laser sources have increased the quality of images as swept-source does not suffer from the sensitivity roll-off of spectral-domain technology and penetrates into deeper layers using infrared wavelengths. This has allowed better imaging of the CC, approaching the resolution of histologic methods [14]. With swept-source OCTA (SS-OCTA) the CC structure and functional perfusion may now be studied in vivo [15].

OCTA imaging of the CC has already allowed for a better understanding of CC changes associated with natural as well as pathologic processes. For instance, the percent of CC area without flow (called flow deficit percentage) in the choriocapillaris has been shown to be significantly affected by age [16, 17]. Specifically, CC flow voids increase with age and the association rises with proximity to the central fovea, with more deficits in the central region [18, 19]. Qualitatively, it has also been shown that CC flow impairment is present in early stages of diabetes sometime even before the appearance of DR on clinical exam, and that long-standing diabetes is correlated with CC drop-out on histology [13, 20]. While there is evidence that CC loss exists in eyes with DR, it is not known whether this degeneration follows a pattern or affects all areas equally. The aim of this study is to examine the relationship between the severity of DR and CC loss. Specifically, it asks how DR severity affects different regions of the CC, given that confounders such as age also affect these regions.

## Methods

### Patient selection

This study included diabetic subjects who were imaged with SS-OCTA in the retina clinic at the New England Eye Center, Boston, MA between January 2017 and July 2018. The study was performed in accordance with the Health Insurance Portability and Accountability Act and adhered to the principles of the Declaration of Helsinki. The subjects signed informed consents and the research was approved by the Institutional Review Board for Tufts Medical Center.

Exclusion criteria comprised patients with a concurrent diagnosis of AMD or other retinal or choroidal vascular disorders, laser in the field of the image, or poor image quality/segmentation. Image quality exclusion was qualitative, rather than based on a signal strength cutoff. Images were graded by an expert grader (author A. Yasin Alibhai MD) into the following groups using fundus imaging and fluorescein angiography when available: diabetes with no diabetic retinopathy (DMnoDR), mild non-proliferative diabetic retinopathy (NPDR), moderate NPDR, severe NPDR, and proliferative diabetic retinopathy (PDR) according to Early Treatment of Diabetic Retinopathy Study (ETDRS) methodology [21]. History of focal laser, pan-retinal photocoagulation (PRP), or anti-vascular endothelial growth factor (anti-VEGF) injection was recorded. Hemoglobin A1c (HbA1c) from within 1 year of imaging was collected.

### Imaging

Images were acquired using the PLEX Elite 9000 SS-OCTA (Carl Zeiss Meditec Inc., Dublin, CA, USA). The PLEX Elite 9000 has an A-scan rate of 100,000 scans per second with a central wavelength of 1060 nm, axial resolution of ~ 6 μm, lateral resolution of ~ 14 μm, and scans at a depth of 3 mm. The standard 6 mm x 6 mm protocol was used, centered on the fovea. This scan protocol consists of 500 B-scans with 500 A-scans per B-scan. The automated segmentation algorithm on the PLEX was used to segment the choriocapillaris slab from 29 µm to 49 µm posterior to the automatically-fitted retinal pigment epithelium line, as is standard on the device [15, 22]. The resulting angiography *en face* image was exported for analysis. Some scans were further excluded due to poor imaging (for example, motion artifact, poor segmentation of the choriocapillaris by the automated algorithm, or laser scar in the field of the image).

### Image processing and analysis

**Fig. 1:**
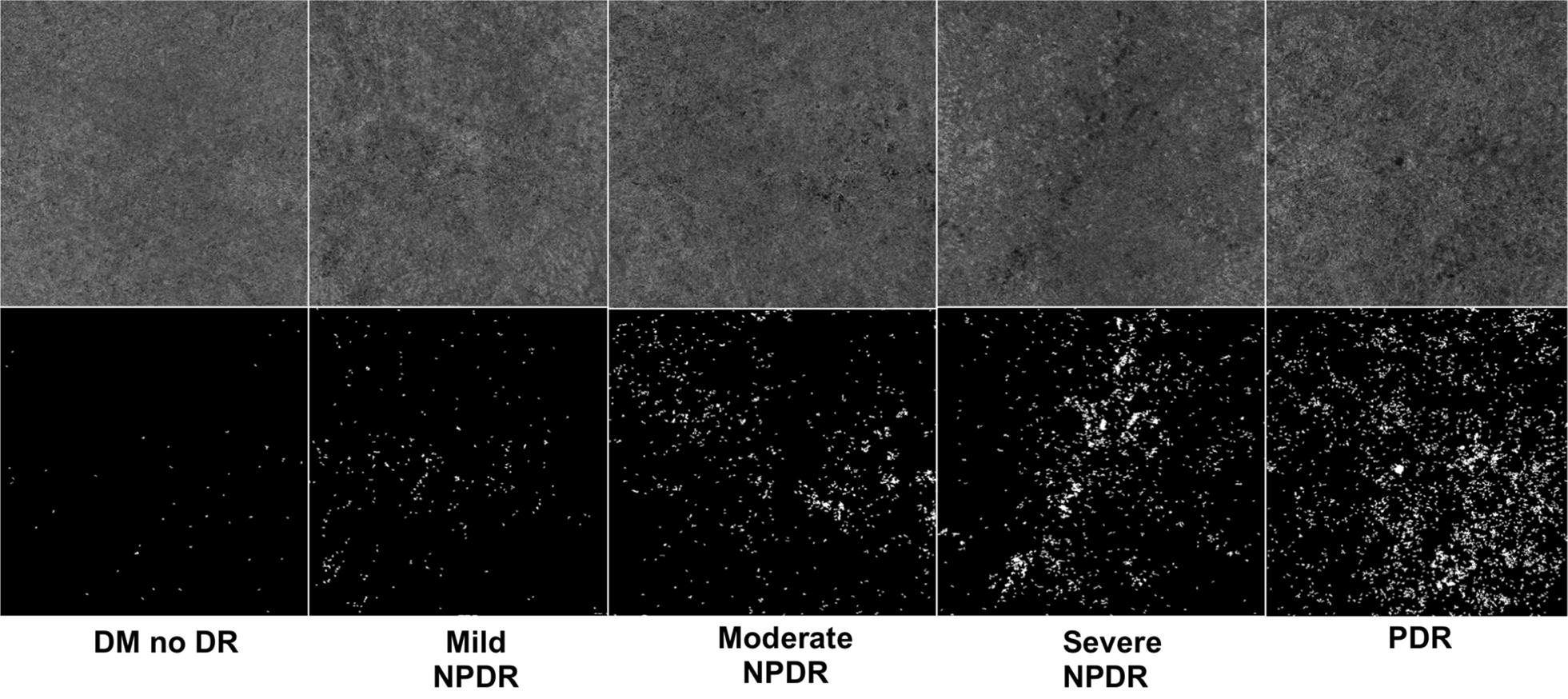
Representative cases for each DR stage with en-face OCTA CC slabs on top and binarized images on the bottom showing flow deficits in white

**Fig. 2:**
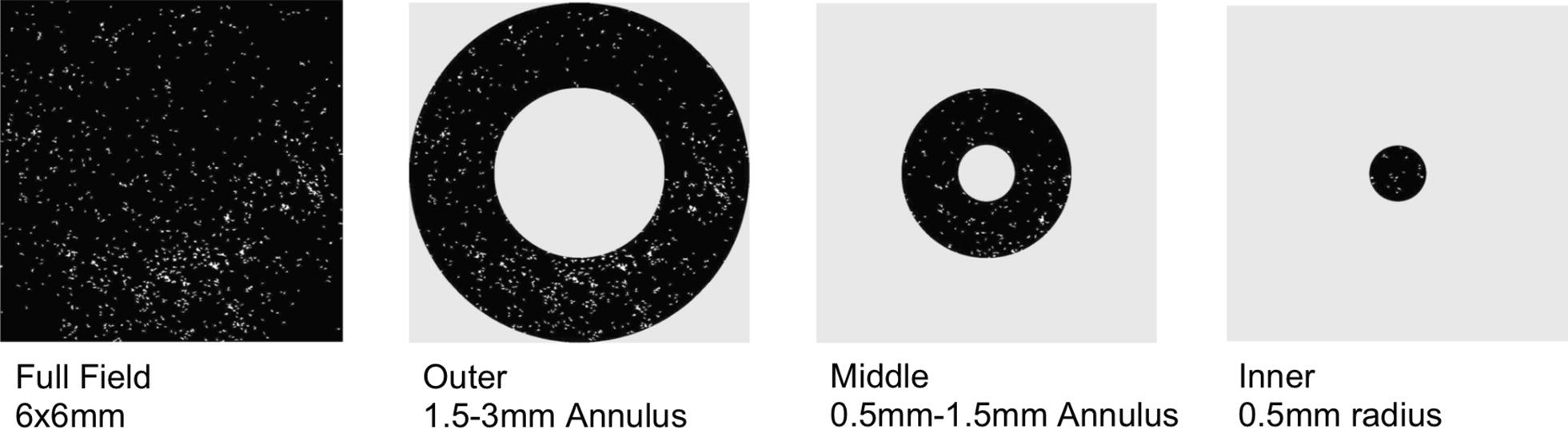
6mm × 6 mm flow deficit percentage regions analyzed with OCTA based on the ETDRS grid

All image processing was performed using a custom Matlab program (MathWorks, Natick, MA, USA). Flow deficit images were computed by binarizing the *en face* OCTA images using a fixed global threshold (threshold value of 0.2, with OCTA data values taking on values in the [0, 1] range); this value was subjectively chosen, independent of this study, on the basis of the qualitative appearance of flow deficits in OCTA data of healthy eyes. Figure 1 depicts representative cases for each DR stage of the binarized images. To reduce the influence of noise, all flow deficits having an area of less than 500 µm^2^ were removed from the analysis. Flow deficit percentage for a given region is calculated as the area of non-flow in the region divided by the total area of the region multiplied by 100. Based on the ETDRS grid, flow deficit percentage was calculated for the Inner (0.5 mm radius circle), Middle (0.5 mm–1.5 mm annulus), Outer (1.5 mm–3 mm annulus) and Full-Field regions for each 6 mm × 6 mm binarized image (Fig. 2). These methods described are similar to those used in the literature [18, 23].

### Statistical analysis

Statistical analysis was done using the open source statistical program R (R Foundation for Statistical Computing, Vienna, Austria) [24]. The specific packages and scripts implemented were *ggplot2* for graphing, *geepack* for GEE analysis, and *MESS* for the quasi-Akaike Information Criterion (QIC) function, which allows for model confirmation [25–27]. Mean FD % in each region by age group and DR severity were calculated. Mean HbA1c was calculated for each severity group. This study included paired eye data from subjects, which introduced dependency in the outcome measured that could be resolved by analyzing the data with a generalized estimating equations (GEE) approach. The GEE analysis is an extension of the generalized linear model that produces a model of the effects, and their significance levels, on a dependent variable (in this case FD %); specifically it is able to take into account correlated data such as paired-eye data. The GEE analysis determined the coefficient and significance of the contribution to the model for the different variables of interest, age and severity [28]. An exchangeable correlation structure was chosen using both a priori assumptions and confirmed using QIC testing [27–29]. Each patient served as a cluster with correlated outcome measures (left eye, right eye). Disease stage was coded as on a Likert scale and was treated as a continuous variable. A p*-*value of .05 was used as a significance cut-off for all applicable measures.

## Results

Out of 186 eyes, 26 eyes were excluded due to poor image quality or coexisting unrelated retinal disease. 160 eyes from 90 diabetic patients with SS-OCTA imaging met inclusion criteria. The mean age was 58.14 years. 51 were male and 39 were female. HbA1c values were available for 71 of the 90 patients. Study population characteristics are outlined in Table 1. Of the total 160 eyes, 19 eyes had a history of PRP, 21 eyes had a history of focal laser with scarring outside of the area of scanning, and 37 eyes had a history of anti-VEGF injection.

**Table 1:** Subject demographic characteristics

DR severity	Pts	Eyes (OD, OS)	Mean age	Male	Female	Mean HbA1c (% pts with HbA1c available)
DM no DR	33	58 (31, 27)	60.33	16	17	7.27 (81.81%)
Mild NPDR	17	31 (17, 14)	60.06	9	8	7.36 (94.11%)
Moderate NPDR	8	15 (7, 8)	65.75	6	2	7.91 (100%)
Severe NPDR	10	16 (7, 9)	54.4	9	1	9.14 (50%)
PDR	22	40 (21, 19)	52.32	11	11	8.75 (68.18%)
Total	90	160 (83, 77)	58.14	51	39	7.83 (78.89%)

**Fig. 3:**
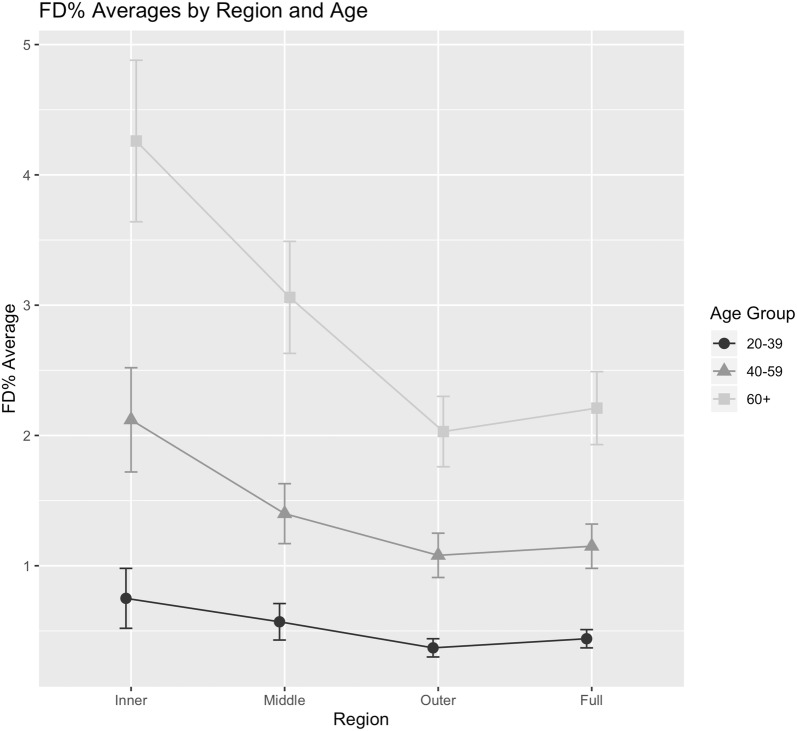
Plot showing flow deficit percentage (FD %) averages of age groups for each region. Error bars represent the standard error of the mean. Data uncorrected for other covariates such as disease severity

**Fig. 4:**
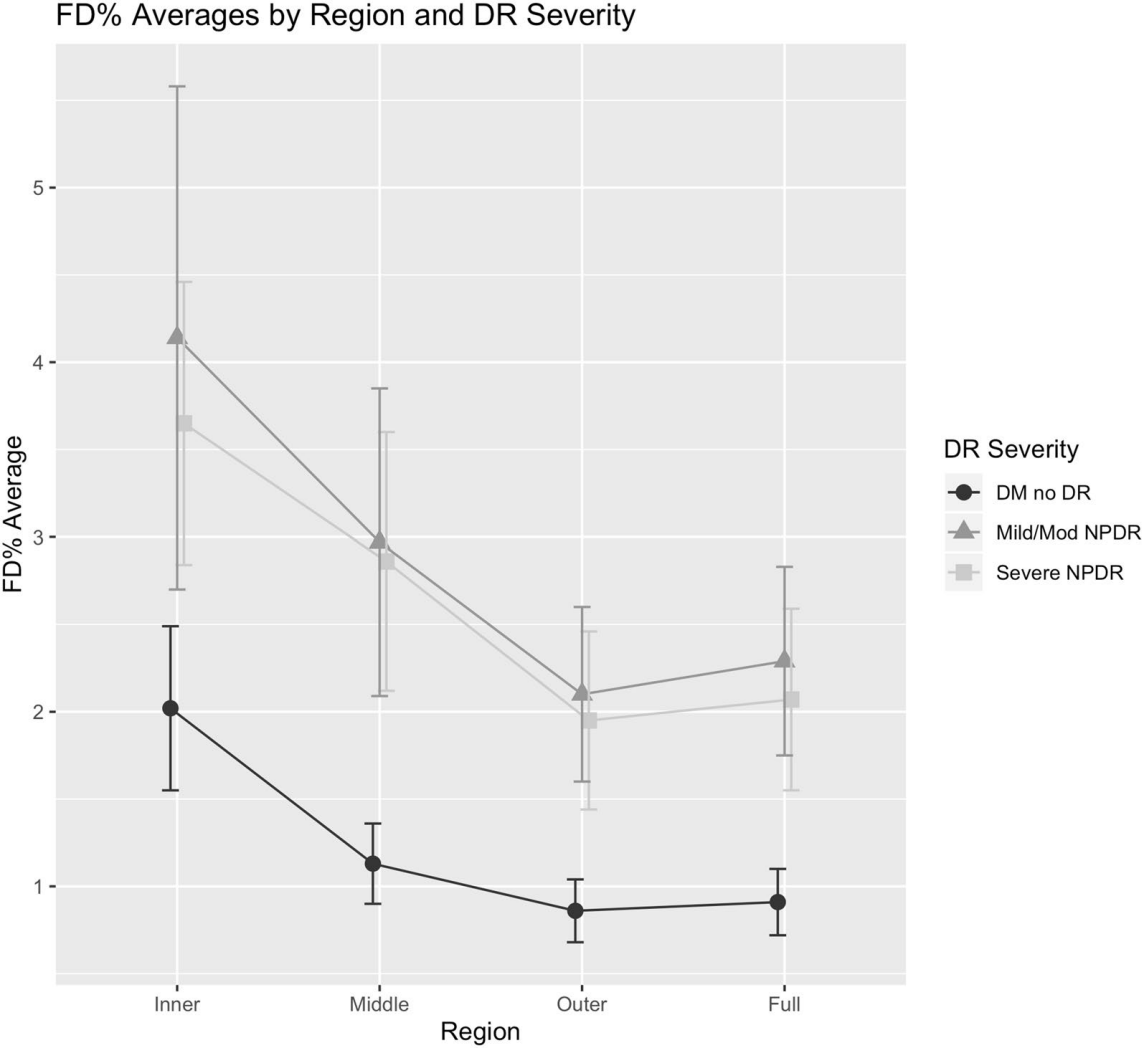
Plot showing flow deficit percentage (FD %) averages of diabetic retinopathy (DR) severity groups for each region. Error bars represent the standard error of the mean. Data uncorrected for other covariates such as age

Using simple averages, uncorrected for other variables, age and DR Severity showed increasing FD % with proximity to the fovea (Figs. 3 and 4). Using the GEE approach, which takes account for paired eye correlation and other included variables, age and DR severity were independently shown to have a significant positive association with FD % with greater effect size in the central two regions (Table 2). The coefficient can be thought of as the amount FD % would increase given a one unit increase in the covariate (age or DR Severity). Other GEE models tested included additional covariates such as gender, history of VEGF injection, history of PRP, history of focal laser, presence of edema, and eye laterality. None of these additional covariates showed significance, in agreement with a previous study [10]. Thus only results for the model including age and DR severity are reported. The α correlation values are non-zero, showing correlation between eyes of the same subject, which justifies using the GEE approach.

**Table 2:** GEE results for the effect and significance of age and DR severity on FD % in the regions studied

Region	Age coefficient (std error)	P value	DR severity coefficient (std error)	P value	α correlation
Inner	0.12 (0.03)	< 0.001	0.65 (0.25)	0.0087	0.29
Middle	0.09 (0.02)	< 0.001	0.56 (0.17)	0.0012	0.29
Outer	0.05 (0.01)	< 0.001	0.33 (0.11)	0.0045	0.35
Full	0.06 (0.01)	< 0.001	0.36 (0.12)	0.0018	0.32

Coefficient is the average increase in FD % for a one unit increase in the covariate (age or DR severity) while controlling for other covariates. The α correlation values are non-zero, showing correlation between eyes of the same subject

## Discussion

Improvements in OCTA technology have only recently allowed for detailed, in vivo study of the CC. This study affirms previous findings of the effects of age and DR on the CC, and offers new insights into the effects of DR severity on flow deficit percentage in the CC. An important tool used in this study was the generalized estimating equation which allows for the use of fellow eyes, increasing the power of the study as well as the ability to identify the significance and effect size of multiple covariates [30].

This study agrees with previous findings in both OCTA and histology that suggest the CC degenerates with age [16–19]. Our results suggest that aging is significantly associated with an increase in flow deficit percentage of the CC, and this finding increases with proximity to the central fovea. The effect of age on FD % in the innermost regions studied is roughly twice that in the outer region and full-field measures. Given that multiple studies have found this effect, it becomes clear that future studies must control for these factors to correctly interpret findings.

While diabetes is a systemic disease, traditionally the ophthalmologic focus has been on its retinal effects. Diabetes as a choroidopathy is relatively under-studied partly because, until recently, detecting changes in the choroidal vasculature was difficult (limited to angiography and histology). OCTA and enhanced depth imaging (EDI) on OCT have extended the ophthalmologist’s view into the choroid [31]. Using OCTA, Choi et al. and Forte et al. reported regions of CC impairment in subjects with diabetic retinopathy [20, 32]. These authors demonstrated that most subjects, even with non-clinical DR, have CC alterations. Dodo et al. found that non-perfusion of the CC is correlated with diabetic retinopathy and visual acuity. The group concluded that choroidal nonperfusion is likely to part of the pathogenesis of decreased vision and is associated with a disrupted photoreceptor layer [33].

This study found that DR severity had a significant positive association with FD %. That is, as diabetic retinopathy worsened there was a corresponding increasing deficit in the CC. As with age, this effect also increased with proximity to the fovea. Thus, for a progression of one stage in severity the flow deficit percentage increased (worsened) for the two inner regions more than the outer and full field regions. As an example, the inner region coefficient size was 1.8 times that of the effect for the full field image (0.65 versus 0.36, respectively).

This study’s results are qualified by several limitations. Drawbacks of this study include its retrospective nature, lack of age-matched controls, and the relative low numbers of patients in the moderate and severe NPDR groups. Additionally, this study’s results are valid for the effect of DR severity but do not provide a granular pairwise analysis between stages. This means that a progression from no DR to mild NPDR is treated the same as a progression from severe NPDR to PDR. The GEE analysis is an extension of the generalized linear model (GLM), but the effect of disease stage may be such that a non-linear model would better capture the relationship between DR severity and FD %. However, the strengths of the GEE allowed for accounting for correlation between paired eyes, as previously discussed. Moreover, a linear model still allows for conclusions to be made about the direction and magnitude of effects. Future studies should address these concerns as well as look prospectively at the effect of treatment (both pharmaceutical and lifestyle modifications) on FD %. Disease duration should also be accounted for as this has been reported to be an even stronger predictor of disease progression and would likely be a significant covariate of FD % as well [34].

## Conclusions

Swept-source optical coherence tomography angiography imaging of diabetic eyes reveals increased flow deficits in the choriocapillaris with increasing proximity to the central macula that correlate with more severe disease. This study also reaffirms previous findings regarding the topographic effects of age on flow deficit in the choriocapillaris.

## Data Availability

The datasets used and/or analyzed during the current study are available from the corresponding author on reasonable request.

## Abbreviations

anti-VEGF: Anti-vascular endothelial growth factor
DR: Diabetic retinopathy
CC: Choriocapillaris
DMnoDR: Diabetes with no diabetic retinopathy
FD %: Flow deficit percentage
HbA1c: Hemoglobin A1c
ETDRS: Early Treatment of Diabetic Retinopathy Study
EDI: Enhanced depth imaging
NPDR: Non-proliferative diabetic retinopathy
OCT: Optical coherence tomography
OCTA: Optical coherence tomography angiography
PRP: Pan-retinal photocoagulation
SS-OCTA: Swept-source optical coherence tomography angiography
GEE: Generalized estimating equations
